# NFAT5-Mediated Signalling Pathways in Viral Infection and Cardiovascular Dysfunction

**DOI:** 10.3390/ijms22094872

**Published:** 2021-05-04

**Authors:** Guangze Zhao, Sana Aghakeshmiri, Yankuan T. Chen, Huifang M. Zhang, Fione Yip, Decheng Yang

**Affiliations:** 1 Department of Pathology and Laboratory Medicine, University of British Columbia, Vancouver, BC V6T 2B5, Canada; guangze.zhao@hli.ubc.ca (G.Z.); mary.zhang@hli.ubc.ca (H.M.Z.); 2 The Centre for Heart Lung Innovation, St. Paul’s Hospital, Vancouver, BC V6Z 1Y6, Canada; sana.1376@yahoo.com (S.A.); chenterry45@gmail.com (Y.T.C.); fione.yip@hli.ubc.ca (F.Y.)

**Keywords:** NFAT5, TonEBP, microRNA, long non-coding RNA, signalling pathway, viral infection, cardiovascular disease, epigenetic modification

## Abstract

The nuclear factor of activated T cells 5 (NFAT5) is well known for its sensitivity to cellular osmolarity changes, such as in the kidney medulla. Accumulated evidence indicates that NFAT5 is also a sensitive factor to stress signals caused by non-hypertonic stimuli such as heat shock, biomechanical stretch stress, ischaemia, infection, etc. These osmolality-related and -unrelated stimuli can induce NFAT5 upregulation, activation and nuclear accumulation, leading to its protective role against various detrimental effects. However, dysregulation of NFAT5 expression may cause pathological conditions in different tissues, leading to a variety of diseases. These protective or pathogenic effects of NFAT5 are dictated by the regulation of its target gene expression and activation of its signalling pathways. Recent studies have found a number of kinases that participate in the phosphorylation/activation of NFAT5 and related signal proteins. Thus, this review will focus on the NFAT5-mediated signal transduction pathways. As for the stimuli that upregulate NFAT5, in addition to the stresses caused by hyperosmotic and non-hyperosmotic environments, other factors such as miRNA, long non-coding RNA, epigenetic modification and viral infection also play an important role in regulating NFAT5 expression; thus, the discussion in this regard is another focus of this review. As the heart, unlike the kidneys, is not normally exposed to hypertonic environments, studies on NFAT5-mediated cardiovascular diseases are just emerging and rapidly progressing. Therefore, we have also added a review on the progress made in this field of research.

## 1. Introduction

The nuclear factor of activated T cells (NFAT5), also known as tonicity-responsive enhancer-binding protein (TonEBP), is a key transcription factor in the *Rel* family, which also includes NFAT1-4 and NFκB [1,2,3]. The members of this family share a *Rel* homology domain (RHD), a unifying characteristic of NFAT proteins that confers a common DNA-binding specificity [4]. Structurally, NFAT5 is a bipartite protein, with a DNA-binding domain in the *N*-terminus and a transactivation domain (TAD) toward the *C*-terminus [5]. In the region upstream of the RHD, NFAT5 harbours all its functional domains related to nucleus–cytoplasm translocation. These domains include a canonical nuclear export signal (NES), a consensus bipartite nuclear localization signal (NLS) and an auxiliary export domain (AED) [6,7], which make the *N*-terminal half of NFAT5 capable of nuclear localization and DNA binding. However, the NFAT5 protein lacks docking sites for phosphatase calcineurin and thus the calcium/calcineurin signalling cascade is dispensable for activation and nuclear localization of NFAT5 [8]. It also lacks the structural domain found in other NFATs required for the formation of cooperative complexes with activator protein-1 (AP-1) (c-Fos and c-Jun) transcription factors [4,9]. Nevertheless, this notion has been challenged by other studies [10,11]. Due to the characteristic of its structure, the *N*-terminal fragment (amino acids 1–472) of NFAT5 can function as a dominant negative mutant [5]. In the *C*-terminal half, there are two long stretches of glutamine residue [7], making up two TADs involved in transcription activation through the phosphorylation of specific amino acid residues by different kinases [12,13,14].

Although NFAT5 was originally identified in the renal medullary cells as a protective factor and plays a central role in maintaining cellular homeostasis against hypertonic stress [5,7], accumulating evidence has revealed its ubiquitous expression in a hypertonic-independent manner in almost all organs, such as the heart, brain and skeletal muscles [15,16,17,18]. The inducers of NFAT5 expression in non-hypertonic environments are largely other cellular stress conditions, such as heat shock, ischaemia, infection, etc. In addition, recent studies have revealed that certain molecular factors, such as microRNAs (miRNA), long non-coding RNA (lncRNA) and epigenetic modifications, are also involved in regulating NFAT5’s expression and activation [14,19]. These physiological and molecular determinants can modulate the NFAT5 gene’s expression levels by controlling its transcription and translation. On the other hand, the altered NFAT5 expression further regulates the transcription of NFAT5 target genes involved in various signal transduction pathways, leading to immune response, cell survival/apoptosis and different pathological conditions. In this review, we will not focus on NFAT5-related immune responses and diseases, for which the readers may refer to recent reviews [20,21]. Instead, we will summarize the recent progress in NFAT5 expression regulated by miRNA/lncRNA targeting, epigenetic modifications and viral infections, as well as the related signal transduction pathways. Since the heart is not regularly exposed to hypertonic fluids like the kidneys, cardiovascular diseases related to NFAT5 expression have not been widely studied in the past and are now an emerging field of research. Thus, we have also added a brief review in this regard.

## 2. Signal Pathways in Hyperosmotic and Non-Hypertonic Conditions

As the only osmosensitive transcription factor in the *Rel* family, NFAT5 expression mediates a number of signal transduction pathways involved in cell survival, proliferation, migration, etc. These functions are determined by its upregulation and phosphorylation. Specifically, under hypertonic conditions, NFAT5 can be increasingly phosphorylated at many amino acid residues including threonine, serine and tyrosine [12]. For example, phosphorylation at Y143 and T135 enhances the nuclear localization of NFAT5 in HEK293 cells and rat renal inner medulla cells [22,23,24]. In contrast, increasing the phosphorylation of NFAT5-S155 and -S158 by a hypotonic condition reduces the nuclear accumulation of NFAT5 [25]. Based on current understanding, the phosphorylation of NFAT5 at certain sites primarily determines its nuclear localization. Nevertheless, the three serine phosphorylation sites in the TAD of NFAT5 are responsible for the activation of NFAT5, since mutations at these sites reduce the transcriptional activity of NFAT5 [26]. However, how these phosphorylation sites directly stimulate NFAT5 activity remains to be elucidated.

After NFAT5 is activated, it can bind the tonicity-responsive enhancer (TonE) site within the promoter of its target genes to initiate transcription. NFAT5 targets various genes under different physiological conditions. Under hypertonic conditions, NFAT5 controls the expression of aldose reductase (AR), betaine/γ-aminobutyric acid transporter 1 (BGT1), the sodium/myo-inositol transporter (SMIT) and the NaCl-dependent taurine transporter (TauT) to directly resist osmotic stress by stimulating the transport of organic osmolytes into the cytoplasm [27]. Under non-hypertonic environments, NFAT5 can participate in inflammatory responses, which are stimulated by various tonicity-independent mechanisms in both hypertonic and isotonic tissues. These stimuli, such as biomechanical stretching, hypoxemia, infection, cytokines and reactive oxygen species, have a positive effect on NFAT5 expression and the activation of immune cells, which leads to the upregulation of NFAT5 target genes such as inducible nitric oxide synthase (iNOS), tumour necrosis factor (TNF), IL-6 and CCL2 [28,29,30].

**Figure 1:**
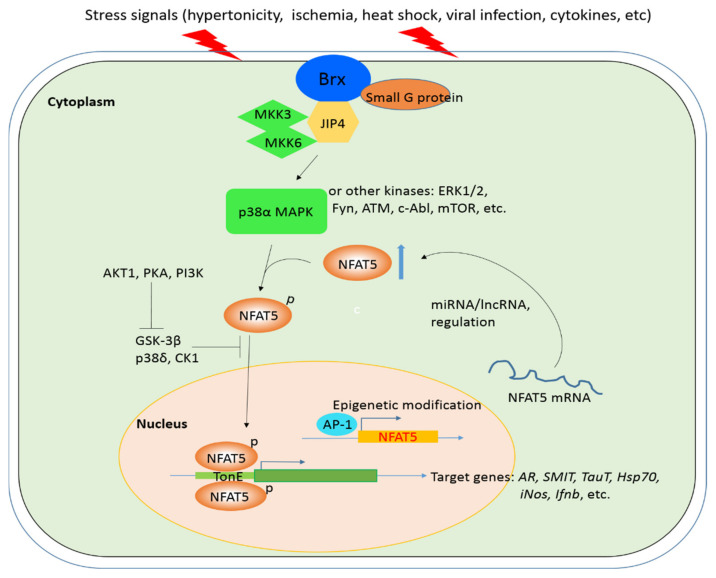
NFAT5-mediated signaling pathways under various cellular stress conditions. The various extracellular stress signals caused by hypertonic or non-hypertonic condition activate signal pathways largely through the Brx, which forms the complex with small G-protein, JIP4 and kinases MKK3/6. The complex then activates p38α-NFAT5 cascade via phosphorylation by MKK3/6. Other kinases can also phosphorylate and activate NFAT5. The phosphorylated NFAT5 translocates into nucleus, binds the TonE site of target gene promoter and initiates transcription of its target genes that response to their corresponding stimuli. Furthermore, GSK-3β, p38δ and CK1 inhibit NFAT5 activation and nuclear localization; however, AKT, PKA and PI3K can suppress the inhibitory effect of GSK-3β on NFAT5 activation by phosphorylation. In addition to kinases, other factors such as miRNA, lncRNA and epigenetic modification also regulate the AP-1-mediated NFAT5 expression and play a role in NFAT5 activation. Thin arrow: promotion; blue thick arrow: upregulation; T-shaped inhibitory arrow: inhibition. Brx: Brevis radix-like proteins; MAPK: mitogen-activated protein kinase; MKK3/6: mitogen-activated protein kinase kinase 3/6; JIP4: JNK-interacting protein 4; ERK/1/2: extracellular signal-regulated kinase1/2; Fyn: Proto-oncogene tyrosine-protein kinase Fyn; ATM: ATM serine/threonine kinase; c-Abl: tyrosine kinases c-Abl; mTOR: mechanistic target of rapamycin; AKT1: RAC-alpha serine/threonine-protein kinase; PKA: protein kinase A; PI3K: phosphoinositide 3-kinases; GSK-3β: glycogen synthase kinase 3β; CK1: casein kinase 1; TonE: tonicity-responsive enhancers; AP-1: activator protein-1; AR: aldose reductase; SMIT: sodium/myo-inositol transporter; TauT: taurine transporter; *Hsp70*: heat shock protein 70; *iNos*: inducible nitric oxide synthase; *Ifnb*: interferon β.

A number of kinases are involved in modulating NFAT5 activity [14]. p38α MAPK, one of the most studied kinases in the pathway, is activated by upstream osmotic stress signals via the activation of Brx, a guanine nucleotide exchange factor (GEF) localized near the plasma membrane [31,32]. Although the precise mechanism by which osmotic stress is sensed by the cell is unclear, it has been suggested that Brx is activated by the stress signal through changes in the cytoskeleton structure [32,33]. Alternatively, Brx may also be activated through changes in its interactions with possible osmosensor molecules or other unknown factors at the cell membrane. Brx activates specific small G proteins (e.g., Rac1/cda-42) through its GEF domain and attracts an intermediate scaffold protein, c-Jun *N*-terminal kinase (JNK)-interacting protein 4 (JIP4). JIP4 then stimulates the p38α MAPK cascade by communicating with its upstream kinases, MKK3 and MKK6 [34]. (Figure 1).

It has been found that NFAT5 expression depends on p38 MAPK [35,36]. However, the underlying mechanism is currently unknown. Since the NFAT5 promoter region contains two activating protein 1 (AP-1)-binding sites and seven interferon-sensitive responsive elements (ISREs) upstream of the transcription start site [31], it is possible that p38 MAPK phosphorylates and activates c-Fos, a component of AP1, and the interferon regulatory factors (IRFs), which bind to AP-1-binding sites and ISREs, respectively, and activate the transcription of the NFAT5 gene [37].

In addition to p38 kinase, other kinases (ERK1/2, Fyn, c-Abl, ATM and mTORC1) can promote the activation of NFAT5 by phosphorylation, which was verified by using specific kinase inhibitors or siRNAs [24,26,38,39,40]. PKC-α, a member of the PKC family of kinases, activates NFAT5 in hypertonic conditions via ERK1/2 [41]; CDK5 can promote NFAT5’s nuclear localization by phosphorylation but has no significant effect on its activity [42]. On the other hand, glycogen synthase kinase 3β (GSK3β), casein kinase 1 (CK1) and p38δ MAPK play a negative role in NFAT5’s nuclear localization and transcriptional activity [25,43,44]. Thus, NFAT5 activity can also be stimulated by PKA, AKT1 and PI3K through their inhibition of the NFAT5-repressive kinase, GSK-3β [43] (Figure 1). Although the Brx-mediated activation of NFAT5 has only been examined in the lymphocyte response to osmotic stress, it is likely that this mechanism is a common one in other cell types [45]. Besides the kinases and other proteins mentioned above, there are additional factors that influence the activation and expression of NFAT5, such as miRNA/lncRNA targeting, epigenetic modification and viral infection, which will be discussed in later sections.

## 3. miRNA/lncRNA-Regulated NFAT5 Expression and Signalling Pathways

The role of miRNAs in gene regulation is largely to suppress translation, which is achieved by targeting the 3′ UTR of mRNA, leading to cleavage or destabilization of the transcripts [46]. LncRNA functions in gene regulation through many mechanisms. One of them is via cross-talking with many other RNAs to serve as a decoy, scaffold and enhancer RNA [47]. LncRNAs that interact with miRNAs are classified as competing endogenous RNAs (ceRNAs) [48]. ceRNAs are also known as miRNA sponges and compete with mRNAs for miRNA binding to modulate gene expression. According to the recent literature, several miRNAs/lncRNAs have been reported to be involved in the regulation of NFAT5 expression and its related signal pathways (Table 1). For instance, miR-466a-3p, miR-20b and miR-30b directly target the 3′ UTR of *Nfat5* mRNA and downregulate its expression [19,49,50]. In a study of miR466a-3p, the authors found that, in transgenic mice, overexpression of miR-466a-3p significantly downregulates the expression of NFAT5 and other osmoregulation-related genes in both the renal cortex and medulla. They concluded that miR-466a-3p and its close relatives (miR-200b and miR-717) are important epigenetic regulators of renal NFAT5 signalling, osmoregulation and urine concentration [49,51]. In a study of miR-20b, it was found that miR-20b directly targets NFAT5 and CAMTA1. However, miR-20b’s expression is downregulated in patients with thymoma-associated myasthenia gravis. The reverse correlation in the expression levels of miR-20b and its target genes suggests that the tumour-suppressive function of miR-20b in thymoma could be due to its inhibition of NFAT signalling by the repression of NFAT5 and CAMTA1 expression [50]. Similarly, miR-30b is another miRNA that directly targets *Nfat5* but is downregulated during pathological conditions. It was found that elevated expression of NFAT5 due to the downregulation of miR-30b in adipocytes leads to the development of obesity and insulin resistance, whereas its ablation enhances adipocyte beiging and prevents ectopic deposition of triglycerides [19].

In addition to these pathogenic effects, studies have also shown miRNA/NFAT5 signalling to be involved in important cellular responses, such as proliferation, differentiation, inflammation and apoptosis. For instance, miR-10b-5p is reported to control myoblast proliferation and differentiation [56]. The authors found that overexpression of miR10b-5p promotes myoblast proliferation and blunted myofiber formation. Further studies revealed that *Nfat5* is a direct target of miR-10b-5p and may serve as a mediator in myogenic differentiation through its downregulation by miR-10b-5p. With regards to studies on T cell proliferation and activation, three groups obtained similar findings associated with the miRNA/NFAT5 signal axis. Li et al. found that miR-568 inhibits the activation and function of CD4+ T cells and T_reg_ cells by targeting *Nfat5* [57]. Serr et al. showed that a miRNA181a-mediated increase in the signal strength of stimulation and co-stimulation links the increased NFAT5 expression with impaired T_reg_ cell induction [58]. The third group demonstrated that a miRNA cluster (miR-106a, -18b and -363-3p) is involved in controlling the differentiation and function of T helper cells by targeting *Nfat5* [59].

Besides the direct regulation of *Nfat5* by miRNA, titin-antisense RNA 1 (TTN-AS1), also known as a tumour promoting lncRNA, could act as a miR411-3p sponge to indirectly regulate cell proliferation and migration. It functions by removing the inhibitory effect of miR-411-3p on NFAT5 to initiate cell growth in oral squamous cell carcinomas [52]. Thus, lncRNA TTN-AS1 contributes to the progression of OSCC via the miR-411-3p/NFAT5 signalling axis. Similarly, the p53/miR-27a/NFAT5 pathway also regulates cell proliferation in mouse ovarian granulosa. Here, p53 inhibits miR-27a, thus eliminating its inhibitory effect on NFAT5 and resulting in cell proliferation through the activation of the Wnt signalling pathway [55]. Another similar study on the Wnt signalling is an investigation of the role of miR-148b in hair follicle growth cycles. It was found that NFAT5 and Wnt10b are the targets of miR-148b. These results demonstrated that miR-148b can activate the Wnt/β-catenin signal pathway by targeting NFAT5 and thus promoting the proliferation of human hair follicle cells [61].

Additionally, several miRNA/NFAT pathways have been shown to be involved in cellular differentiation and macrophage polarization. One of the key regulators of cementoblast differentiation is the miR-361-3p/NFAT5 axis, which is associated with many important signalling pathways including ERK1/2, JNK, p38, PI3K/Akt, NF-κB and Wnt/β-catenin [54]. Furthermore, miR-223 is required for PPARγ-dependent macrophage alternative activation. PPARγ can control miR-223’s expression by directly binding to the PPARγ regulatory elements located in the miR-223 promoter. In addition, RASA1 and NFAT5 are genuine targets of miR-223 and are important for the PPARγ/miR-223 regulatory axis in controlling macrophage alternative activation [60].

Recent literature also points to the crucial role of miRNAs and NFAT5 in other important processes such as angiogenesis, inflammation and apoptosis. For instance, the miR-29c-3p/NFAT5 axis was reported to be associated with the occurrence of Parkinson’s disease [65]. Studies showed that miR-29c-3p inhibits pro-inflammatory cytokine secretion, and NLRP3 inflammasome and NF-κB activation in activated microglia. *Nfat5* is a direct target of miR-29c-3p that can reverse this effect of miR-29c-3p. Thus, miR-29c-3p may attenuate inflammatory responses in activated microglia through the suppression of the NLRP3 inflammasome by targeting *Nfat5*. However, miR-29c-3p was downregulated in this experimental setting, which provided a better understanding of the pathogenesis of Parkinson’s disease. Another important signalling pathway related to apoptosis and inflammation is the colon cancer-associated transcript-1 (CCAT1)/miR-218/NFAT5 axis. CCAT1 is a lncRNA and acts as a neuroprotective agent in astrocytes following ischaemic injury by enhancing cell viability and halting apoptosis. This is achieved by inhibiting miR-218 and, in turn, upregulating its downstream target, *Nfat5,* thereby alleviating inflammation and apoptosis [53].

Certain miRNAs do not directly target *Nfat5* but rather the related genes in the signal cascade. For example, overexpressed miR-30e-5p targets MAP4K4 in HBV-infected cells to inactivate MAPK and thereby suppresses NFAT5 activation. Since NFAT5 is a suppressor of the oncogene DARS2, this suppression promotes HCC tumorigenesis via DARS2 expression [64]. Similarly, miR-338-3p also does not target *Nfat5*; instead, it targets EGF-like domain 7 (EGFL7), a gene potentially promoting tumour cell growth. However, lncRNA SBF2-AS1 (SBF2 antisense RNA 1), which is upregulated by NFAT5 in glioblastoma cells, can sponge miR-338-3p to release the inhibition of EGFL7 and subsequently stimulate angiogenesis in tumours. Thus, NFAT5 contributes to pathogenesis via the SBF2-AS1/miR-338-3p/EGFL7 signalling axis [63]. Another miRNA associated with glioblastoma is miR-641. This miRNA acts as a physiologic inhibitor of the host gene, AKT2, by targeting several kinases (directly or indirectly via NFAT5) that determine the phosphorylation state of AKT2, a proto-oncogene in the PI3/AKT pathway [66]. In human glioblastoma multiforme, this negative feedback loop is disrupted. While AKT2 expression is increased, miR-641 is significantly suppressed. This suppression results in increased expression of PIK3R3 and NFAT5 mRNA, and, secondly, of PDK2, thereby enhancing AKT2 phosphorylation and further supporting malignancy [62].

In summary, the role of NFAT5 miRNAs and their lncRNA sponges is crucial for the regulation of many cellular processes. Please refer to Table 1 for more information regarding these miRNA-regulated signalling pathways and their consequences.

## 4. Epigenetic Regulation of NFAT5

Epigenetic modifications often refer to histone modifications and DNA methylations that regulate gene expression and chromatin architecture without altering the genetic code [67]. These modifications—comprising acetylation, methylation, phosphorylation and ubiquitination, among others—change the accessibility of DNA to the transcription machinery and thus affect gene expression. Consequently, they have been implicated in multiple diseases comprising cancer, metabolic disorders, inflammation, neurological disorders, etc. [68]. According to the recent literature, DNA methylation and histone modification are the two most prominent areas of study associated with the transcription factor NFAT5. Hence, we mainly focus on the two aforementioned epigenetic mechanisms in this review.

### 4.1. NFAT5 and DNA Methylation

DNA methylation constitutes the addition of a methyl group to the DNA molecule, thereby affecting DNA–protein interactions and influencing gene expression. The most prominent DNA methylation in eukaryotes is the addition of a methyl group to the 5′-position of cytosine by enzymes called DNA methyltransferases (DNMTs) [69]. Although it is context-dependent, DNA methylation at the promoter region in particular is typically associated with gene silencing [70]. Recent studies suggest that NFAT5 can repress the expression of other genes by recruiting DNMTs to their promoters. For example, NFAT5 can regulate thermogenesis and obesity by recruiting DNMT1 to the promoter of b3-adrenoceptor, a key regulator in thermogenesis and obesity, resulting in the suppression of thermogenesis and the beiging of white adipose tissue, leading to obesity and insulin resistance [19].

The methylation status of the *Nfat5* gene can be altered under different conditions; for instance, during HBV infection, NFAT5 expression is suppressed by inducing hypermethylation at the AP1-binding site of its promoter [71]. Similarly, in osteoarthritis, the *Nfat5* promoter was hypermethylated in the cartilage specimens collected from patients [72]. With the DNA immunoprecipitation technique, *Nfat5* was found to contain several differentially methylated regions in the T cells and monocytes of adult males displaying physical aggression compared with the control group [73]. Hence, *Nfat5* can not only be regulated by methylation at its own promoter region under different conditions, but it can also regulate other genes through the recruitment of DNMTs to their promoters. For instance, the target gene expression of NFAT5 can also be controlled by the methylation status. Johansen et al. found that the NFAT5 binding sites on its target genes were hypermethylated and repressed in female patients with glioma. This, in turn, is associated with survival advantages [74].

### 4.2. NFAT5 and Histone Modification

Modifications to the histone proteins, such as methylation, acetylation, phosphorylation and ubiquitination, can regulate gene expression. These modifications not only affect DNA accessibility but also act as distinct codes to recruit different proteins and thus affect cell function [75]. Recent studies suggest that NFAT5 can influence histone methylation and acetylation under different conditions.

NFAT5 is known to play a crucial role in mediating the immune response induced by Toll-like receptors in macrophages [20]. An important component of this response is the induction of the dynamic remodelling of chromatin architecture. Buxadé et al. found that recruitment of NFAT5 to target genes such as *Nos2* requires de novo protein synthesis and chromatin remodelling, which is sensitive to histone deacetylase (HDAC) inhibitors; this is because inhibition of HDAC with its inhibitor trichostatin A (TSA) suffices to induce the association of NFAT5 with target genes whose expression requires chromatin remodelling. Although it cannot be ruled out that TSA can cause other effects in addition to inhibiting histone deacetylation, this result supports the concept that inhibition of HDACs may favour the recruitment of NFAT5 to genes such as *Nos2* [28]. Furthermore, in a study focusing on adipocytes, the authors found that NFAT5 suppresses the activation of the PPARλ2 promoter by binding to its upstream region. This binding is associated with di-methylation of Lysine 9 in histone H3 and limits chromatin access. In response to adipogenic signals, NFAT5 dissociates from the promoter, leading to the opening of the locus, in association with reduced histone H3 methylation and recruitment of the transcription factor C/EBPβ. Reduced NFAT5 expression results in a profound enhancement of PPARγ 2 expression and increased adipogenesis [76].

NFAT5 expression has differential effects on histone acetylation under different conditions. For instance, under hypertonic stress, a condition that benefits NFAT5 expression and activation, NFAT5 can induce histone hyperacetylation at the aldose reductase (AR) locus that spans the 5′ upstream sequences and the exons of the AR gene. This finding indicates that NFAT5-induced histone acetylation has a role in AR gene activation through chromatin remodelling [77]. On the contrary, under hypoxic stress, overexpression of NFAT5 diminishes histone acetylation in astrocytes through the activation of the Sirtuin 1 (SIRT1)/ nuclear factor-E2 related factor (NRF2) pathway, which protects astrocytes against oxygen-glucose-serum deprivation/restoration damage [78].

## 5. NFAT5-Associated Activities and Signalling Pathways during Viral Infection

While NFAT5 is widely known to be involved in hypertonic stress responses, there is substantial evidence that illustrates its role in modulating viral infections (Table 2). To date, a large number of signalling molecules and transcription factors have been shown to modulate HIV-1 gene expression through interactions with the HIV-1 long terminal repeat (LTR) enhancer region, especially in dendritic cells and cells of the monocyte/macrophage lineage. Pereira et al. published a list of binding sites and their corresponding transcription factors that interact with the HIV-1 promoter. This list included NFAT proteins, NFκB and many others [79]. The authors indicated that these biding sites and their relative orientations within the LTR mediate combinational DNA–protein and protein–protein interactions that form a complex regulatory signal network through which HIV-1 regulates its levels of positive- and negative-sense gene expression under a variety of extracellular stimuli. Ranjbar et al. identified the positive role of NFAT5 in the replication of HIV-1 in human monocyte-derived macrophages (hMDMs) [80]. Specifically, NFAT5 was demonstrated by the authors to bind a conserved site in the LTR enhancer region of HIV-1 subtypes B, C and E, and promote viral propagation. Impressively, this unique NFAT5 binding site is also conserved in HIV-2 and in simian immunodeficiency virus (SIV) from multiple primate species. Additionally, with small interfering RNAs (siRNAs) being used to knock down the expression of endogenous NFAT5 in HIV-1 subtypes B-, C- or E-infected hMDMs, the production of HIV-1 was significantly inhibited, suggesting the ability of the siRNAs to suppress HIV-1 replication. Later, Ranjbar’s group further reported the crucial role of NFAT5 in *Mycobacterium tuberculosis* (MTb)/HIV-1 co-infected human peripheral blood cells and monocytes [81]. MTb is known to induce viral replication and stimulate immune signalling pathways, leading to increased mortality in patients infected with HIV-1. Strikingly, the authors noticed that the silencing of NFAT5 inhibited MTb-stimulated HIV-1 replication in co-infected macrophages, and identified a NFAT5 binding site in the viral promoter of the HIV-1 subtype B and subtype C genomes, which is required for efficient induction of HIV-1 replication by MTb. Moreover, the group also found that MTb can induce the expression of NFAT5 via the MyD88-dependent signalling pathway, while knocking down MyD88 efficiently inhibits MTb-induced expression of NFAT5. In addition, two MyD88-associated adaptors, IRAK1 and TRAF6, were also shown to be necessary for MyD88’s upregulation of NFAT5.

As NFAT5’s expression is known to modulate cell survival and apoptosis, recent studies have often focused on its pathogenic role in multiple cancers, including virus-associated carcinoma. For instance, hepatitis B virus (HBV) infection plays an essential role in the development of hepatocellular carcinoma (HCC). Qin et al. revealed that NFAT5 serves as a tumour suppressor for HCC by downregulating the expression of DARS2, an oncogene that promotes the proliferation of HCC cells [64]. Confirming this with a chromatin immune-precipitation (ChIP) assay and next-generation sequencing, the authors demonstrated that NFAT5 negatively regulates the expression of DARS2 by directly targeting its promotor. On the other hand, as discussed earlier, HBV infection inhibits NFAT5 expression through the miR-30e-5p/MAP4K4 signalling axis, leading to the upregulation of DARS2 and, in turn, the progression of hepatocarcinogenesis.

Hepatitis C virus (HCV) was also demonstrated to promote its own propagation through a NFAT5-dependent signalling pathway. The non-structural 5A (NS5A) protein of HCV plays multifunctional roles in cellular signal transduction and hepatic diseases such as HCC. Lim et al. found that the protein level of heat shock protein 72 (Hsp72) is raised in cells expressing HCV NS5A protein [82]. Furthermore, NFAT5 levels are also significantly increased in a NS5A stable cell line as compared with vector-transfected control cells. Next, the authors found that the elevation of Hsp72 in NS5A stable cells is dependent on the induction of NFAT5, which was further verified by functional analysis using specific siRNAs. Together, these findings suggest that NS5A regulates *Hsp72* via NFAT5, leading to the enhancement of HCV replication.

Coxsackievirus B3 (CVB3) is the predominant pathogen associated with myocarditis. During coxsackievirus infection, a number of cellular host proteins are cleaved by CVB3 proteases, leading to the modulation of various cellular signalling pathways. In our studies, we first showed that NFAT5 is upregulated in non-immune cells, such as HeLa cells and a human cardiomyocyte cell line, during CVB3 infection. We also found that NFAT5 is cleaved by CVB3 protease 2A at the amino acid position G503 and produces a *N*-terminal fragment that plays a dominant negative role in NFAT5 activity [83]. We further revealed its antiviral activity on CVB3, since the knockdown of NFAT5 enhances viral replication. Specifically, NFAT5 induces the activation of iNOS, a downstream target of NFκB, by collaborating with NFκB. Furthermore, iNOS exhibits an inhibitory effect on CVB3 replication and decreases cardiomyocyte injury. Our in vivo experiments further showed that hypertonic treatment of mice by peritoneal administration of hypertonic saline at the time of CVB3 infection enhanced iNOS expression and had a protective antiviral effect in the heart [83].

Vesicle stomatitis virus (VSV) was shown to be resistant to high salt conditions via mediation of the p38MAPK/ATF2/AP-1 signalling pathway [85]. Although the authors did not measure the levels of NFAT5 expression, the hypertonic condition in this experimental setting surely played a role in driving antiviral type I interferon (IFN-I) signalling in macrophages. Indeed, the authors demonstrated that a high-salt diet protected mice from lethal VSV infection through IFN-I signalling in the macrophages. IFN-I is a well-studied cytokine that enhances efficient antiviral immunity and prohibits viral replication within target cells. However, the role of IFN-I is a double-edged sword, as its excessive expression can elicit destructive inflammatory responses and cause haematopoietic stem cell (HSC) exhaustion. A recent study identified NFAT5 as a transcriptional suppressor of virus-induced IFN-I production [84]. This is evidenced by the fact that a key interferon regulatory factor 3 (IRF3)-dependent activator element in the *Ifnb1* enhanceosome contains an overlapping binding site for NFAT5, which opposes IRF3 and thus balances IFNß expression. Toll-like receptor 3 (TLR3) is an important pattern recognition receptor (PRR) that activates IFN-I. The authors found that NFAT5 not only binds the *Ifnb1* enhanceosome, but also suppresses *Ifnb* promoter activity in a TLR3-dependent manner. In addition, the authors noticed enhanced IFN-I production and viral clearance upon lymphocytic choriomeningitis virus (LCMV) infection in mice lacking *Nfat5*. Furthermore, infection of *Nfat5*-deficient macrophages (BMDMs) and GM-CSF-induced conventional bone marrow-derived myeloid DCs (GM-BMDCs) with VSV or murine CMV (MCMV) also showed higher expression levels of IFNB1. These findings illustrate that NFAT5 limits the magnitude of the IFN-I response in vivo and has a significant impact on both antiviral defence and maintenance of HSC quiescence [84].

## 6. NFAT5 Signalling in Cardiovascular Dysfunction

Despite the fact that NFAT5 is ubiquitously expressed throughout the entire body, its role in the heart, which is not exposed to hypertonic fluid like the kidneys, has been less studied. Importantly, recent studies have indicated that NFAT5 may be a key biomarker in the heart, with its expression levels being directly related to disease development or recovery [86,87]. This section summarizes the roles of NFAT5 and the respective signalling responses involved in several cardiovascular disorders (Table 3).

### 6.1. Cardiomyocyte Cytotoxicity

NFAT5 has been documented to protect against cytotoxicity in cardiomyocytes. Specifically, NFAT5 regulates the promoter region of the *TauT* gene, which maintains a high concentration of the cardioprotective molecule taurine in the heart [88,89]. Doxorubicin is a very potent anticancer agent, but its use is limited by its dose-dependent, irreversible cardiotoxicity. In studies of the mechanism of doxorubicin-induced cytotoxicity, Ito et al. demonstrated that doxorubicin promotes the cardiomyocyte-specific ubiquitin-independent degradation of NFAT5 by proteasome-mediated proteolysis. Depletion of NFAT5 subsequently downregulates TauT expression in the cardiomyocytes [88]. In ischaemia-induced cytotoxicity, taurine is protective, as it inhibits the ubiquitin-dependent proteasomal degradation of NFAT5 [89]. Furthermore, taurine promotes nuclear entry of NFAT5 to upregulate TauT expression and prevent cell death. Both doxorubicin and ischaemia-induced cardiotoxicity involve NFAT5 activation, which suppresses reactive oxygen species formation and increases cell survival in cardiomyocytes [88,89]. To minimize doxorubicin-induced cytotoxicity, recent studies have focused on the role of Sirtuin 1 (SIRT1), a nicotinamide adenosine dinucleotide (NAD+)-dependent deacetylase, in the protection of the heart from the cytotoxic injury. The revealed mechanism is likely to be related to the activity and deacetylation of SIRT1’s downstream target proteins [90]. Thus, clinical application of the SIRT1 agonists may be a strategy to reduce doxorubicin-induced cardiotoxicity.

### 6.2. Myocardial Infarction

Anisosmotic changes in cardiomyocytes occur during infarction-induced ischaemia and reperfusion. As a tonicity-responsive transcription factor, NFAT5 helps cells adapt to altered osmotic stress and plays a protective osmoregulatory role in the heart. By exposing cultured cardiomyocytes to various tonic conditions, Navarro et al. elucidated the mechanism of bidirectional regulation of NFAT5 activity, where hypertonicity activated and hypotonicity repressed NFAT5’s transcriptional activity [91]. In the signalling pathway, hypertonicity induces NFAT5 activity by increasing its mRNA and protein expression, which upregulates the expression of its downstream target genes, such as *AR* and *Hsp70* [91].

On the contrary, NFAT5 can also be detrimental following myocardial infarction. Arctigenin, a cardioprotective compound, was found to inhibit NFAT5 in cardiac macrophages [92]. More specifically, arctigenin binds the DNA-binding domain of NFAT5, thus inhibiting its ability to facilitate the NFκB signalling pathway and suppressing the conversion of macrophages to an inflammatory phenotype. Since the inflammatory macrophage phenotype is associated with increased infarct size during postinfarction modelling, NFAT5 is suggested to exacerbate postinfarction cardiac injury.

### 6.3. Arterial Wall Stress

Hypertension causes biomechanical stretching stress in the arterial walls, which drives maladaptive remodelling, causing arterial walls to stiffen and malfunction. Thus, this structural cardiovascular abnormality can damage various organs, including the heart, kidneys, brain and other vasculatures, and lead to premature morbidity and death [100,101,102]. Mechanistically, biomechanical stretching in vascular smooth muscle cells (VSMC) increases NFAT5 protein production through activation of the c-Jun *N*-terminal kinase while also inducing NFAT5’s nuclear translocation [11]. The nuclear translocation of NFAT5 requires palmitoylation by carnitine palmitoyltransferase family 1 [11,93]. Using a cDNA microarray for screening and a ChIP assay for confirmation, the same studies also identified the potential downstream targets of NFAT5, namely tenascin-C, which is associated with detrimental arterial remodelling, and κ-actin, which is involved in VSMC migration. Based on their findings, the authors suggested that another key transcription factor, AP-1, regulates *Nfat5* transcripts by binding to multiple AP-1 binding sites on the *Nfat5* promoter [11] (Figure 1).

The role of NFAT5 in arterial wall remodelling was also studied by using an inducible smooth muscle cell (SMC)-specific *Nfat5* knockout mouse model [94]. In cultured mouse VSMCs, loss of the *Nfat5* gene inhibited the expression of gene sets involved in controlling the cell cycle, and the interaction with the extracellular matrix and cytoskeletal dynamics. In vivo, SMC-specific knockout of *Nfat5* did not affect the general vascular architecture and blood pressure levels under baseline conditions. However, proliferation of VSMCs and the thickening of the arterial wall wee inhibited during both flow-induced collateral remodelling and hypertension-mediated arterial hypertrophy. These findings identify NFAT5 as a novel molecular determinant of biomechanically induced phenotype changes in VSMCs and wall stress-induced arterial remodelling processes [94].

### 6.4. Atherosclerosis

Relating to the previously mentioned VSMC migration, the role of NFAT5 in atherosclerosis is a current area of investigation. Movement of migratory VSMC into the arterial lumen is a characteristic of atherosclerosis. Depending on the environmental signals, NFAT5 can respond to either angiotensin II or platelet-derived growth factor-BB (PDGF-BB) to transform VSMC into a contractile or migratory phenotype, respectively [95]. Through a luciferase reporter assay, NFAT5 was shown to bind the first intron of the smooth muscle alpha actin (*SMɑA*) gene and to be necessary for angiotensin II-stimulated conversion to the contractile phenotype. For the migratory phenotype, migration and proliferation assays demonstrated that NFAT5 is only required for PDGF-BB-induced VSMC migration and not for VSMC proliferation. Additional in vivo data showed that *Nfat5* is upregulated in the artery following acute vascular injury and in chronic atherosclerosis.

With regards to the immune cells, chemotactic migration of macrophages into the atherosclerotic lesion is triggered by macrophage colony-stimulating factor (M-CSF) and requires NFAT5 [96]. This previous finding is in line with a recent study using the rat hindlimb ischaemia model, which demonstrated that gene silencing of *Nfat5* attenuates arteriogenesis and angiogenesis in rats. The authors also found that NFAT5 can regulate monocyte recruitment by regulating the expression of monocyte chemoattractant protein 1 (MCP-1) in endothelial cells, both in vivo and in vitro, and that MCP-1 supplementation can reverse the inhibitory effect of *Nfat5* knockdown on monocyte recruitment and arteriogenesis. Collectively, these findings suggest that NFAT5 promotes arteriogenesis via MCP-1-dependent monocyte recruitment [97].

A more recent pathway of atherosclerosis pertains to the activation of NFAT5 by hypertonicity [98]. NFAT5 enhances the transcription of the NLR family pyrin domain containing 3 (NLRP3) inflammasome, which is followed by NFAT5 mediating interleukin 1β transcription to augment inflammation in endothelial cells and recruit monocytes [98]. Another downstream factor, vascular endothelial growth factor C (VEGF-C), is upregulated by NFAT5 to stimulate atherosclerotic lesion progression by inducing intimal neovascularization [95]. However, VEGF-C also appears to be protective against salt-induced hypertension. In mononuclear phagocyte system cells, NFAT5 responds to a high-salt diet by triggering VEGF-C secretion from macrophages [99]. The authors demonstrated that VEGF-C triggers lymph capillary network modification for the extrarenal regulation of interstitial osmolarity. Additionally, VEGF-C stimulates endothelial NOS production, a known protective factor for blood pressure homeostasis.

## 7. Conclusions and Perspectives

The accumulated evidence has verified that NFAT5 is not only regulated by tonicity but can also be stimulated by various tonicity-independent factors in both hypertonic and isotonic tissues. For this reason, its roles in many diseases, such as rheumatoid arthritis, diabetes mellitus, cancer, cardiovascular disease and brain disorders, have been studied. The data demonstrated that NFAT5 is a critical transcription factor in immunity and in autoimmune diseases. In addition, these studies have also shown that tonicity-independent stimulation of NFAT5 expression is critical for its tissue-specific functions, such as enhanced cell survival, migration, proliferation, vascular remodelling and angiogenesis. However, under various pathological conditions, the influence of NFAT5 expression on disease induction has not been thoroughly studied yet, particularly in the characterization of the mechanisms in a tissue-specific environment. Open questions include which target genes are regulated by NFAT5 in certain disease conditions of a given organ and which signal transduction pathways are involved in this disease induction and progress. Although some new regulators of NFAT5 expression, such as miRNA/lncRNA, epigenetic modification and viral infection, have been identified recently, their further roles in NFAT5 activation and pathogenesis need to be further elucidated. Currently, a number of kinases have been suggested to be responsible for NFAT5 phosphorylation and its activity [14]; however, which kinase plays a critical role in NFAT5 activation is unclear and the knowledge of phosphorylation’s effect on NFAT5 transactivation activity has fallen short of explaining how NFAT5 is transported between cytoplasmic and nuclear compartments. In addition, the interaction between NFAT5 and other transcription factors, e.g., c-Fos and c-Jun, is still unclear [4,10,103]. This implies that the components of the transcriptional complex required for NFAT5-dependent gene transcription remain elusive. Structurally, NFAT5 lacks the calcineurin-binding domain, and thus it is widely accepted that calcium/calcineurin signalling is dispensable for NFAT5 activation. However, some studies have reported that calcineurin is needed for the expression of osmotic-stress-inducible NFAT5-regulated genes in mouse CD4 T cells in a high-salt medium [104]. This finding suggests that calcineurin and NFAT5 communicate to a certain level and may do so through specific post-translational modifications, which need to be clarified.

Previous studies largely used mouse or rat models to analyse the impact of NFAT5 deficiency in different scenarios to support its role in autoimmune-associated inflammatory diseases and osmoregulatory disorders. Future work may consider some osmotic-stress-independent disorders such as cardiovascular disease and viral infections. With the development of stem cells and CRISPR/Cas9 technologies, studies using human iPS cell-derived primary cells containing specific mutations in the human genome will shed new light on the role of NFAT5 in human health and diseases.

## Figures and Tables

**Table 1:** miRNAs and lncRNAs involved in regulation of NFAT5 expression and signalling pathways.

Cell Type/Disease	miRNA/lncRNA	Consequence	Reference
Adipocytes	miR-30b	miR-30b negatively regulates NFAT5. Upregulation of NFAT5 expression by knockdown of miR-30b contributes to the development of obesity and insulin resistance.	[19]
Renal medulla	-miR-466a-3p -miR-200b & miR-717	miR-466a-3p, miR-200b and miR-717 downregulate NFAT5 expression during osmotic response. High level of miR-466a-3p is associated with polydipsia, polyuria and disturbed ion balance.	[49,51]
HEK293 cells and human thymoma tissue	miR-20b	miR-20b contributes to the suppression of thymoma and thymoma-associated myasthenia gravis and inhibits T-cell activation and proliferation. The tumor suppressive function of miR-20b is via inhibiting NFAT5 expression.	[50]
Oral squamous cell carcinoma	miR-411-3p/lncRNA TTN-AS1	The lncRNA Titin antisense RNA 1 (TTN-AS1) acts as a miR-411 sponge, thereby inhibiting the miR-411 which is a negative regulator of NFAT5. Overexpression of NFAT5 restores cell growth in TTN-AS1 depleted cells.	[52]
Astrocytes	miR-218/lncRNA CCAT1	miR-218 targets NFAT5. The lncRNA colon cancer-associated transcript-1 (CCAT1) acts as a miR-218 sponge, thereby activating NFAT5 expression, which is crucial for controlling apoptosis and inflammation.	[53]
Cementoblasts	miR0361-3p	Overexpression of miR-361-3p suppresses cemantoblast differentiation through directly targeting NFAT5.	[54]
Mouse Ovarian granulosa	miR-27a	NFAT5 promotes cell proliferation through activating the Wnt signaling pathway. miR-27a directly inhibits NFAT5 expression. However, p53 negatively regulates miR-27a. Hence p53/miR-27a/NFAT5 pathway regulates mouse granulosa cell proliferation.	[55]
Myoblasts	miR-10b-5p	Knockdown of NFAT5 represses myoblast differentiation. miR-10b-5p regulates C2C12 myoblast differentiation and proliferation by directly targeting NFAT5 and repressing its activity.	[56]
T cells	-miR-106a, miR-18b and miR-363-3p -miR-181a -miR-568	A miRNA cluster of miR-106a, miR-18b and miR-363-3p is involved in the differentiation and function of T helper cells through directly inhibiting NFAT5. miR-568 affects the activation and function of CD4+ T cells and T_regs_ through targeting NFAT5. miR-181a enhances NFAT5 activation axis and is involved in regulating T-cell induction and autoimmunity linked to type 1 diabetes.	[57,58,59]
Macrophage	miR-223	PPARγ/miR-223 regulatory axis controls macrophage polarization through targeting downstream target genes such as NFAT5 and RASA1.	[60]
Sheep Wool follicle	miR-148b	miR-148b positively regulates proliferation of hair follicles through activating Wnt/β-catenin signalling pathway and inhibiting NFAT5.	[61]
Human primary glioblastoma	miR-641	miR-641 is tumor suppressive and negatively regulates the PI3K/Akt pathway via directly targeting several kinases and indirectly targeting NFAT5. miR-641 is downregulated in glioblastoma.	[62]
Glioblastoma cell & glioma samples	miR-338-3p/lncRNA SBF2-AS1	NFAT5 upregulates SBF2-AS1, which can sponge miR-338-3p, a negative regulator of EGFL7. Thus, NFAT5 promotes glioblastoma cell-driven angiogenesis via the SBF2-AS1/miR-338-3p/EGFL7 signaling pathway.	[63]
Hepatoma	miR-30e-5p	miR-30e-5p targets MAP4K4 to inactive MAPK and thereby suppresses NFAT5, which leads to promotion of HCC tumorigenesis via the oncogene DADS2 expression.	[64]
Microglia	miR-29c-3p	miR-29c-3p suppresses inflammasome activation via targeting NFAT5, impairing inflammatory response in Parkinson’s disease.	[65]

**Table 2:** Altered NFAT5 expression and activation of signalling pathways during viral infection.

Virus	Cell Type/Disease	Altered NFAT5 Expression and Consequences	Reference
HIV	HeLa-CD4 cells, THP-1 cells, human MDMs/AIDS	HIV infection itself does not cause any changes in NFAT5 mRNA levels. NFAT5 binds to the long terminal repeat enhancer region conserved in HIV-1, HIV-2 and multiple SIVs to promote viral propagation.	[80]
HIV/MTb	Human PBMCs, human MDMs/AIDS	MTb and HIV co-infection upregulates NFAT5 expression via the MyD88-dependent signalling pathway. NFAT5 promotes MTb-stimulated HIV-1 replication by binding to the viral promoter of HIV-1 subtypes B, C and E to form a complex regulatory signal network.	[81]
HBV	HCC tissues from patients, hepatocytes/HCC	HBV inhibits NFAT5 expression through the miR-30e-5p/MAPK4K signalling axis. NFAT5 serves as an HCC tumour suppressor by downregulating the expression of the oncogene DARS2.	[64]
HCV	NS5A stable cell lines/HCC	Overexpression of HCV NS5A upregulates NFAT5, which benefits HCV replication via an increase in heat shock protein 72.	[82]
CVB3	HeLa cells, SV40 immortalized human cardiomyocytes/myocarditis	NFAT5 is upregulated early after infection, which inhibits CVB3 replication through the induction of iNOS production, and is then cleaved by viral proteases, which benefits viral pathogenesis.	[83]
LCMV	NFAT5-deficient mouse model	NFAT5 supresses the production of IFN-1 via binding to the promoter region of the *Ifnb1* gene and limiting the recruitment of IRF3. IFN-1 production and viral clearance were enhanced upon LCMV infection in NFAT5-deficient mice.	[84]
VSV/MCMV	Macrophages, dendritic cells	*Nfat5* mRNA accumulates and reaches a maximal expression at 24 h in macrophages infected with VSV or MCMV. IFN-1 responses are repressed in VSV- and MCMV-infected macrophages and dendritic cells.	[84]

**Table 3:** Altered NFAT5 expression and cardiovascular dysfunction.

Cardiovascular Dysfunction	Cell Type or Model	Altered NFAT5 Expression and Consequences	Reference
Dox-induced cytotoxicity	Cardiomyocytes	Dox promotes NFAT5 degradation, leading to downregulated TauT expression and cardiomyocyte injury.	[88,89,90]
Myocardial infarction	Cultured cardiomyocytes, cardiac macrophages	Hypertonicity upregulates NFAT5 to induce downstream target gene expression; NFAT is involved in activating macrophages to exacerbate postinfarction damage.	[91,92]
Arterial wall stress	VSMC, mice	Biomechanical stretching upregulates NFAT5 to influence downstream target genes, such as tenascin-C and κ-actin, in arterial remodelling and VSMC migration.	[11,93,94]
Atherosclerosis	VSMC, mice	NFAT5 converts VSMC to the contractile and migratory phenotypes after ANG II and PDGF-BB stimulation, respectively.	[95]
	Macrophages	NFAT5 is involved in macrophage chemotactic migration by M-CSF stimulation.	[96]
	Human umbilical vein endothelial cells, rat	NFAT5 promotes arteriogenesis and angiogenesis by MCP-1 monocyte recruitment.	[97]
	Mouse, human umbilical vein endothelial cells and monocytes	NLRP3 inflammasome activation by NFAT5 increases IL-1b expression to facilitate inflammation in endothelial cells and recruit monocytes.	[98]
	Macrophages	VEGF-C upregulation is protective against salt-induced hypertension and stimulates eNOS to protect blood pressure homeostasis. VEGF-C upregulation stimulates intimal neovascularization and enhances atherosclerotic lesion progression.	[95,99]
